# Role of RNA modifications in carcinogenesis and carcinogen damage response

**DOI:** 10.1002/mc.23418

**Published:** 2022-05-13

**Authors:** Michelle Verghese, Emma Wilkinson, Yu‐Ying He

**Affiliations:** ^1^ Department of Medicine, Section of Dermatology University of Chicago Chicago Illinois USA; ^2^ Pritzker School of Medicine University of Chicago Chicago Illinois USA; ^3^ Committee on Cancer Biology University of Chicago Chicago Illinois USA

**Keywords:** arsenic, carcinogenesis., chemical carcinogens, DNA damage response, environmental carcinogens, epitranscriptomics, m^6^A, metal, virus

## Abstract

The field of epitranscriptomics encompasses the study of post‐transcriptional RNA modifications and their regulatory enzymes. Among the numerous RNA modifications, *N* ^6^‐methyladenosine (m^6^A) has been identified as the most common internal modification of messenger RNA (mRNA). Although m^6^A modifications were first discovered in the 1970s, advances in technology have revived interest in this field, driving an abundance of research into the role of RNA modifications in various biological processes, including cancer. As analogs to epigenetic modifications, RNA modifications also play an important role in carcinogenesis by regulating gene expression post‐transcriptionally. A growing body of evidence suggests that carcinogens can modulate RNA modifications to alter the expression of oncogenes or tumor suppressors during cellular transformation. Additionally, the expression and activity of the enzymes that regulate RNA modifications can be dysregulated and contribute to carcinogenesis, making these enzymes promising targets of drug discovery. Here we summarize the roles of RNA modifications during carcinogenesis induced by exposure to various environmental carcinogens, with a main focus on the roles of the most widely studied m^6^A mRNA methylation.

## 1 INTRODUCTION

RNA species, including messenger RNA (mRNA), transfer RNA (tRNA), ribosomal RNA (rRNA), and noncoding RNAs (ncRNAs), can be patterned with over 100 different post‐transcriptional modifications, collectively referred to as the epitranscriptome.^1^, ^2^ Functionally, RNA modifications regulate gene expression by modulating RNA metabolism.^3^ RNA modifications play a role in many biological processes, including development, the cellular stress response, aging, and diseases such as cancer.^4^, ^5^, ^6^

In cancer, recent studies have demonstrated that RNA modifications and their associated regulatory enzymes are dysregulated, the effects of which are context‐dependent.^4^, ^7^ Although many cancers result from exposures to carcinogens, studies on the roles of RNA modifications during carcinogenesis from exogenous exposures are limited. It is important to consider how specific carcinogens may differentially regulate RNA modifications during cancer initiation, as new insights into these early mechanisms are critical for developing specialized therapies.

In this review, we summarize evidence for the role of RNA modifications in promoting carcinogenesis in response to specific metal, chemical, inhalation, dermal, and viral exposures. We focus primarily on carcinogens, which are classified as carcinogenic to humans (Group 1), as well as agents that are classified as probably (Group 2A) or possibly carcinogenic (Group 2B) to humans by the International Agency for Research on Cancer (IARC) and for which RNA modifications have been shown to have a potential role in promoting malignancy.^8^

## 2 RNA MODIFICATIONS AND THEIR ENZYMES

The development of sensitive detection methods and high‐throughput RNA‐sequencing technologies has advanced the characterization and mapping of RNA modifications.^9^, ^10^ The most extensively studied RNA modification is *N* ^6^‐methyladenosine (m^6^A) on mRNA. Other modifications on mRNA described here include 1‐methyladenosine (m^1^A), 5‐methylcytosine (m^5^C), and N6, 2′‐O‐dimethyladenosine (m^6^Am).^11^

**Figure 1:**
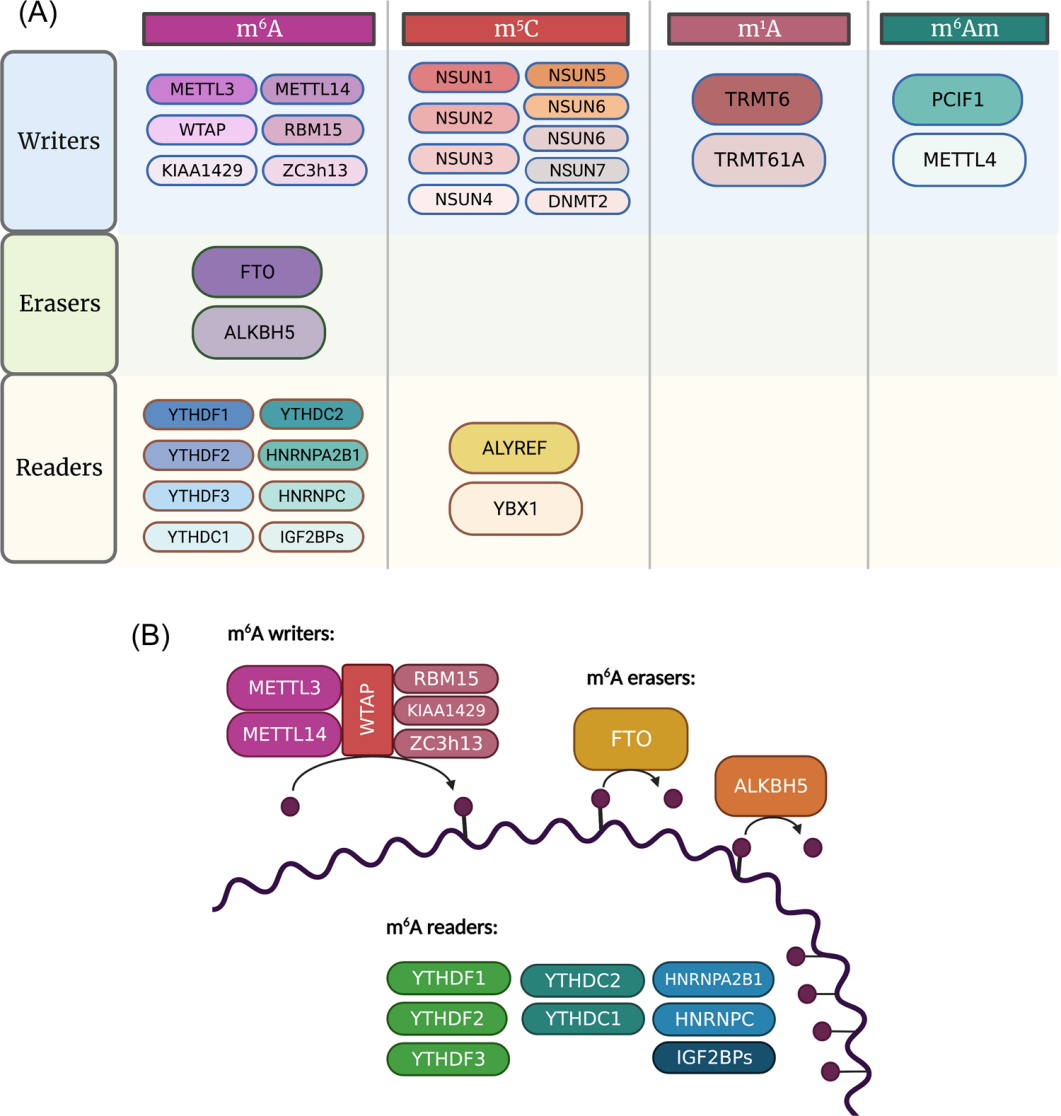
RNA modifications and their regulatory enzymes. (A) Writers, erasers, and readers of *N* ^6^‐methyladenosine (m^6^A), 5‐methylcytosine (m^5^C), 1‐methyladenosine (m^1^A), and N6, 2′‐O‐dimethyladenosine (m^6^Am). (B) The m^6^A writer complex catalyzes the addition of m^6^A modifications to RNA. Erasers mediate their removal, whereas m^6^A readers detect the modifications.

Regulation of the epitranscriptome is mediated by three categories of regulatory enzymes: writers, erasers, and readers. Writers catalyze the addition of the modification to the RNA molecule; erasers catalyze the removal of the modifications.^12^ Readers serve to recognize the modifications (Figure 1).^12^ The coordination of these enzymes can drive molecular processes including transcription, RNA export, translation, and decay.^3^, ^9^, ^12^

### 2.1 m^6^A

m^6^A methylation is the most prevalent internal modification in eukaryotic mRNA. It is a reversible modification conserved across various organisms, including mammals, plants, viruses, and bacteria.^13^, ^14^, ^15^, ^16^ m^6^A is found on roughly 0.1–0.4% of total adenosine nucleotides in mammals.^17^, ^18^ Studies into the methylome landscape using m^6^A‐seq and next‐generation sequencing have revealed that m^6^A is found in over 7000 human genes and is enriched in long internal exons, near stop codons, and at the 3′‐untranslated region (UTR).^10^, ^19^

m^6^A is deposited by a multicomponent writer complex, comprising methyltransferase‐like family members 3 and 14 (METTL3 and METTL14), and Wilms‐tumor 1‐associated protein (WTAP).^20^ This complex interacts with other proteins, including RBM15, KIAA1429, and ZC3H13.^20^, ^21^, ^22^, ^23^ m^6^A erasers, or demethylases, include fat mass and obesity associated (FTO) prand Alkb homolog 5 (ALKBH5).^24^, ^25^ m^6^A readers include YTH‐binding proteins 1, 2, and 3 (YTHDF1, YTHDF2, and YTHDF3), YTH domain‐containing 1 and 2 (YTHDC1 and YTHDC2), heterogeneous nuclear ribonucleoprotein A2/B1 and C (HNRNPA2B1 and HNRNPC), and IGF2BPs.^26^, ^27^

### 2.2 m^5^C

m^5^C is found on both tRNA and mRNA at the 5′‐ and 3′‐UTRs.^28^ m^5^C writers include the human ortholog NOP2/Sun domain protein 2 (NSUN2) and other NSUN family members, NSUN1‐7, as well as DNA methyltransferase homolog (DNMT2).^29^, ^30^ m^5^C readers include the Aly/REF export factor (ALYREF) and Y‐box‐binding protein 1 (YBX1).^31^, ^32^

### 2.3 m^1^A

m^1^A modifications are less abundant than m^6^A, constituting about 0.02% of adenosine in human mRNA, and are preferentially enriched around the start codon.^33^, ^34^ m^1^A writers include tRNA Methyltransferases 6 and 61a (TRMT6 and TRMT61a).^35^

### 2.4 m^6^Am

m^6^Am modification consists of a dimethylation of the adenosine nucleoside.^36^ m^6^Am is found adjacent to the mRNA cap structure at the transcription start nucleotide and internally on U2 small nuclear RNA (snRNA); it is estimated that 50–80% of starting adenosine nucleotides contain an m^6^Am modification.^37^ The writers of m^6^Am are phosphorylated CTD interacting factor 1 (PCIF1) and METTL4.^38^, ^39^

## 3 CARCINOGEN EXPOSURES

### 3.1 Metal exposures

Evidence suggests that exposures to toxic metals arsenic, cadmium, and nickel can cause carcinogenesis by modulating cellular pathways through RNA modifications. Further studies are needed to determine whether exposure to other toxic metals, such as cobalt, lead, and chromium, can regulate RNA modifications to drive cancer development.

#### 3.1.1 Arsenic

**Figure 2:**
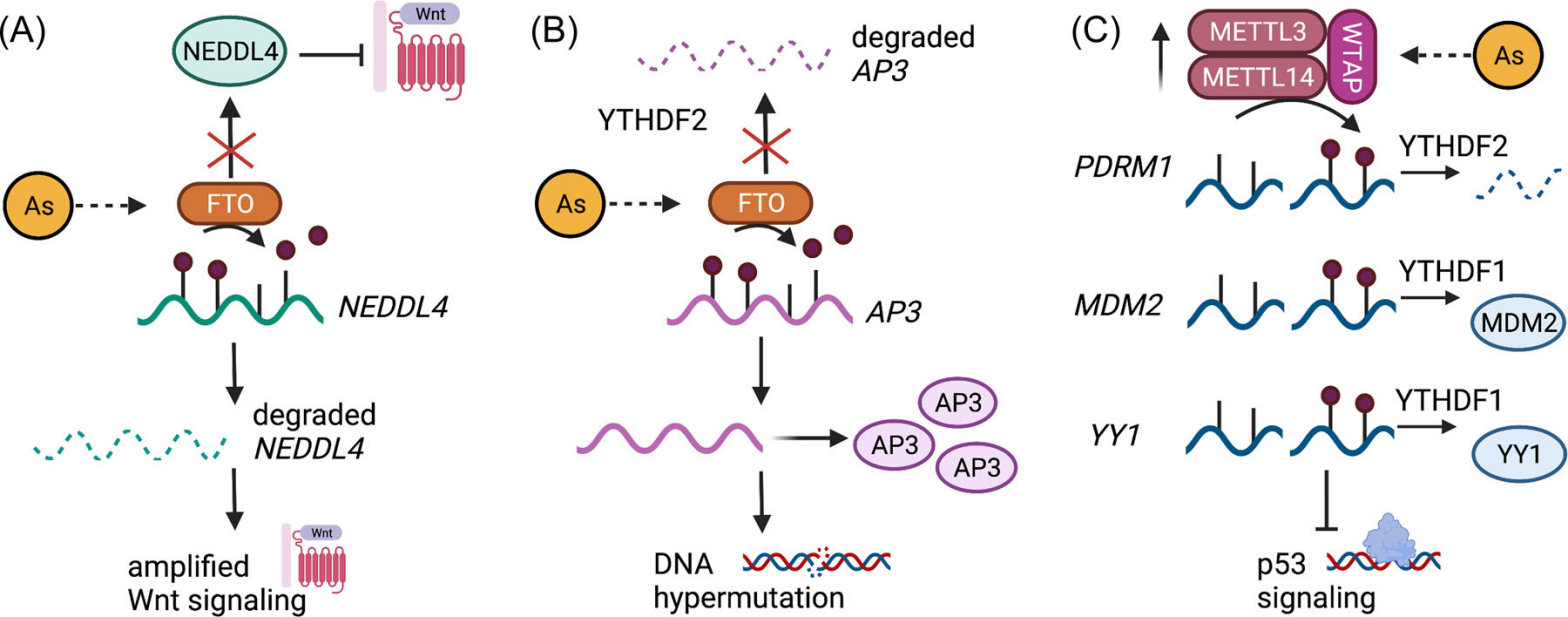
Proposed molecular mechanisms of arsenic‐induced carcinogenesis. (A) Upregulation of fat mass and obesity associated (FTO) results in demethylation and degradation of *NEDDL4* transcripts, promoting Wnt signaling. (B) Upregulation of FTO demethylates AP3 transcripts to increase AP3 translation, causing DNA hypermutation. (C) Upregulation of the m^6^A methyltransferase complex results in degradation of PDRM1 and upregulated expression of MDM2 and YY1, leading to inhibited p53 signaling.

Arsenic is a naturally occurring metalloid in Earth's crust and an IARC Group 1 carcinogen.^40^ Exposure to inorganic arsenic through contaminated drinking water is associated with skin, lung, bladder, and liver cancer.^41^ Several studies have found that exposure to arsenic resulted in decreased m^6^A levels (Figure 2). Cui et al.^42^ showed that in human keratinocytes, chronic exposure to relevant low levels of arsenite (100 nM) for 28 weeks increased FTO levels through arsenic‐mediated inhibition of p62‐dependent selective autophagy, resulting in decreased global m^6^A levels.^42^ This study also found that the tumor‐suppressive ubiquitin ligase *Nedd4l* was a gene target of FTO in this context.^42^ Decreased m^6^A on the *Nedd4l* transcript decreased *Nedd4l* mRNA stability, leading to the activation of Wnt signaling and tumorigenesis.^42^ Other studies have found decreased global m^6^A levels in arsenic‐treated adenocarcinomic human alveolar basal epithelial (A549) cells.^43^, ^44^ Gao et al.^43^ found that treatment with 2 μM of arsenic decreased global m^6^A mRNA levels by upregulating FTO in A549 cells. In particular, this study found that FTO demethylated transcripts of *Apobec3* (*A3B*), a DNA deaminase and key driver of arsenic‐induced mutagenicity.^43^ This demethylation increased *A3B* expression by inhibiting YTHDF2‐mediated *A3B* mRNA decay, leading to DNA damage and hypermutation in arsenic‐induced carcinogenesis.^43^ Cayir et al.^44^ also found decreased global m^6^A levels in arsenic‐exposed A549 cells, although the mechanism was not investigated in this study.

In contrast, Zhao et al.^45^ found that keratinocytes treated with 1 μM of arsenite for 5 months showed increased m^6^A levels, increased expression of METTL3, METTL14, WTAP, KIAA1429, and YTHDF1, and decreased expression of FTO. In particular, elevated m^6^A methylation of the p53 activator PRDM2 and p53 inhibitors YY1 and MDM2 was observed.^45^ YTHDF2‐mediated decay of PRDM2 transcripts, together with YTHDF1‐mediated upregulation of YY1 and MDM2 translation, resulted in the inhibition of the tumor suppressor p53 and keratinocyte transformation.^45^ Similarly, in human bronchial epithelial (HBE) cells treated with 2.5 μM arsenite for 13 weeks, m^6^A levels and expression of METTL3, METTL14, and WTAP expression were increased, whereas FTO was decreased.^46^ Furthermore, another study found that NSUN2 expression was decreased after arsenite treatment in mouse keratinocytes, resulting in loss of tRNA methylation.^47^

#### 3.1.2 Cadmium

Cadmium is a heavy metal and IARC Group 1 carcinogen, and is strongly associated with the development of pulmonary cancer.^48^ Cadmium exposure is primarily occupational.^48^ Yang et al.^49^ found that cadmium‐induced transformation of SV‐HUC‐1 human uroepithelial cells increased overall m^6^A levels and increased m^6^A deposition on cancer‐promoting genes, including *Cdcp11*, which encodes for an oncogenic transmembrane glycoprotein. Mechanistically, METTL3 deposited m^6^A onto the 3′‐UTR of *Cdcp1* mRNA, resulting in increased CDCP1 expression through a YTHDF1‐dependent mechanism in transformed cells.^49^ Similarly, cadmium‐treated HBE (BEAS‐2B) cells displayed decreased m^6^A levels, along with increased expression of ALKBH5.^50^ ALKBH5 was found to demethylate mRNA of the tumor suppressor *Pten*, decreasing *Pten* mRNA stability and reducing PTEN expression, which promoted cadmium‐induced cell transformation.^50^ In addition, in a study of cadmium‐induced oxidative damage, pancreatic β‐cells exposed to cadmium sulfate displayed decreased levels of m^6^A, METTL3, and FTO, although the precise nature of this relationship was not elucidated.^51^

#### 3.1.3 Nickel

Nickel is an IARC Group 1 carcinogen that can cause cancers of the lung, nasal cavity, and paranasal sinuses.^52^ Yang et al.^49^ found that nickel‐induced transformation of SV‐HUC‐1 human uroepithelial cells resulted in increased m^6^A levels and increased m^6^A deposition on *Cdcp1* mRNA, causing increased CDCP1 translation in a YTHDF1‐dependent manner.

#### 3.1.4 Other metals

Chromium, cobalt, and lead are toxic heavy metals and IARC carcinogens.^52^, ^53^, ^54^ These heavy metals are found in contaminated soil and water; occupational exposures also occur during industrial processes.^52^, ^53^, ^54^ Few studies have assessed changes in RNA modifications after these exposures. In HEK293T cells, exposure to chromium for 24 h altered levels of inosine modification on mRNA and upregulated expression of the adenosine deaminase ADAR1 that catalyzes the A‐to‐I modification.^55^, ^56^ Additionally, in the cortexes of C57BL/6 mice exposed to cobalt for 30 days, decreased m^6^A levels were noted, coupled with increased expression of FTO and ALKBH5, and decreased expression of METTL3, METTL14, and WTAP.^57^ Functionally, the effects of cobalt exposure in this context were associated with neurodegenerative damage.^57^ Furthermore, in adolescent C57B16 mice exposed to lead for 20 days, the m^6^Am writer METTL4 was downregulated; however, changes in m^6^Am levels were not evaluated.^58^

### 3.2 Chemical exposures

There is evidence that exposure to chemical carcinogens, such as Fumoisin B1 (FB1) and diethylnitrosamine, can modulate RNA modifications to promote carcinogenesis by affecting oncogenic cellular pathways. Current work on arecoline exposure suggests a role for RNA modifications in promoting existing cancers, but not in inducing cancer initiation. Lastly, recent studies into other chemical carcinogens, including aflatoxin B1 (AFB1), carbon nanotubes (CNTs), cyclophosphamides (CYPs), and endocrine‐disrupting chemical exposures, indicate that these carcinogens can modulate RNA modifications and influence other biological processes, but how these changes may promote cancer initiation is unknown (Table 1).

**Table 1:** Summary of RNA modifications in response to various chemical exposures

Carcinogen	IARC group	Cells or organisms	Finding	Biological process	References
Fumoisin B1	2B	HepG2 cells	↑m^6^A	Oxidative stress and hepatocarcinogenesis	^60^
			↑METTL3, ↑METTL14, ↑YTHDF1, ↑YTHDF2, ↑YTHDC2, ↑YTHDF3		
			↓FTO, ↓ALKBH5		
Diethylnitrosamine	2A	C57Bl/6N	↑FTO	HCC carcinogenesis	^62^
		mice			
Arecoline	1	SCC25 and CAL27‐A cells	↑FTO, ↑METTL3	OSCC proliferation	^65^
Arecoline	1	OECM1, SAS, and CGHNC9 cells	↓ YBX1	OSCC	^66^
Aflatoxin	1	BME cells	↓m^6^A,	Cytotoxicity	^69^
			↓METTL3, ↓METTL14,		
			↑ALKBH5		
Aflatoxin	1	C57BL/6J mice	↑m^6^A, ↑METTL3,	Oxidative stress	^70^
			↓FTO, ↓YTHDF2		
Aflatoxin	1	HepaRG cells	↓TRMT6	–	^71^
Aflatoxin	1	Caco‐2 cells	↑ALYREF	Colon cytotoxicity	^72^
Carbon nanotubes	2B	HBE cells (16HBE14o‐)	↓m^6^A,	–	^75^
			↑ hm5c		
Carbon nanotubes	2B	C57BL/6 mice	↑TRMT61A	–	^76^
Cyclophosphamide	1	Neonatal rat cardiac myocytes	↑m^6^A,	Cardiotoxicity	^79^
			↑METTL3		
Cyclophosphamide	1	hGCs and mouse ovary	↑m^6^A,	Ovarian damage	^80^
			↑METTL3, ↑METTL14, ↑Zc3h13, ↑KIAA1429,		
			↓FTO,		
			↓YTHDF1,		
			↓YTHDF2,		
			↓YTHDC1,		
			↓YTHDC3		
PCB	1	C57BL/6J mice	↑m^6^Am,	Nonalcoholic steatohepatitis	^83^
			↑PCIF1		
PCB	1	Human primary blood mononuclear cells	↓WTAP,	–	^84^
			↓RBM15		
DHEP	2B	Sprague–Dawley rats	↑m^6^A,	Testicular injury	^86^
			↑YTHDC2,		
			↓FTO		
Triclosan	–	Zebrafish larvae	↓m^6^A,	Obesity and nonalcoholic fatty liver disease	^90^
			↑YTHDC1,		
			↑YTHDF1,		
			↑FTO		
Bisphenol‐A	–	Zebrafish larvae	↓m^6^A,	Obesity and nonalcoholic fatty liver disease	^90^
			↑YTHDC1		
			↑YTHDF1		
Bisphenol‐A	–	A549 cells	↓m^6^A	–	^44^
Vinclozolin	–	A549 cells	↓m^6^A	–	^44^

*Note*: Refer to original reference for full list.

Abbreviations: ALKBH5, Alkb homolog 5; BME, bovine mammary epithelial; DHEP, Di(2‐ethylhexyl)phthalate; FTO, fat mass and obesity; HCC, hepatocellular carcinoma; hGCs, human ovarian granulosa cells; hm^5^C, 5‐hydroxymethylcytosine; METTL3, methyltransferase‐like family member 3; METTL14, methyltransferase‐like family member 14; m^6^A, *N* ^6^‐methyladenosine; PCIF1, phosphorylated CTD interacting factor 1; OSCC, oral squamous cell carcinoma; WTAP, Wilms‐tumor 1‐associated protein; YBX1, Y‐box‐binding protein 1; YTHDC1, YTH domain‐containing 1; YTHDC2, YTH domain‐containing 2; YTHDF1, YTH‐binding protein 1; YTHDF2, YTH‐binding protein 2; YTHDF3, YTH‐binding protein 3.

#### 3.2.1 FB1

FB1 is a toxin produced by Fusarium molds found primarily in maize products and was considered possibly carcinogenic to humans (IARC Group 2B) after causing hepatocellular carcinoma (HCC), cholangiocarcinoma, and renal tubule carcinomas in mouse models.^59^ Increased m^6^A levels were observed in HepG2 cells exposed to FB1 for 24 h, coupled with increased expression of writers METTL3 and METTL14, decreased expression of erasers FTO and ALKBH5, and increased expression of readers YTHDF1, YTHDF3, YTHDC2, and YTHDF2. The transcription factor NRF2 promotes an antioxidant response upon FB1 exposure and an increase of m^6^A‐modified *Nrf2* transcripts upregulated NRF2 expression via a YTHDF1, YTHDF3, and/or YTHDC2 mechanism in response to FB1.^60^ In contrast, expression of KEAP1, an NRF2 inhibitor, was decreased in response to FB1 as FB1‐induced increases in m^6^A on the *Keap1* transcript resulted in decreased *Keap1* expression via a YTHDF2 mechanism.^60^ This suggests that the KEAP1‐NRF2 antioxidant response is activated in response to FB1‐induced reactive oxygen species and it is hypothesized that the prolonged activation of NRF2 signaling may be a mechanism by which FB1 promotes hepatocarcinogenesis.^60^

#### 3.2.2 Diethylnitrosamine

Diethylnitrosamine, or *N*‐Nitrodiethylamine (DEN), is a synthetic oil and hepatocarcinogen classified in Group 2A, and is found in gasoline and other industrial materials.^61^ In a study of tumor initiation in HCC, male mice injected with 100 mg/kg DEN had elevated *Fto* mRNA and protein expression at 24 and 48 h postinjection.^62^ CUL4A is an oncogene associated with HCC; in DEN‐exposed mice, *Cul4a* transcripts were demethylated by FTO, resulting in decreased *Cul4a* expression.^62^ This suggests a protective role for FTO against DEN‐induced HCC development.^62^

#### 3.2.3 Arecoline

Arecoline is an alkaloid and IARC Group 1 carcinogen found in the areca nut, and chewing the areca nut is linked to a high risk of oral cancer due to arecoline exposure.^63^, ^64^ In oral squamous cell carcinoma (OSCC) cell lines treated with 1 μM arecoline for 90 days, FTO and METTL3 protein expression were increased.^65^ The same study also found that FTO promoted cancer development and proliferation of arecoline‐transformed OSCC by maintaining cancer stemness and mediating cisplatin resistance.^65^ In another study of arecoline‐induced OSCC, oral cancer cell lines treated with areca nut extract for 3 months showed decreased expression of the m^5^C reader YBX1.^66^

#### 3.2.4 AFB1

AFB1 is a mycotoxin which can pollute grains and animal products.^67^ AFB1 is metabolized to AFM1 after consumption by cows and can contaminate dairy products such as milk.^67^ AFB1 is classified as an IARC Group 1 carcinogen and it is associated with liver and breast cancers.^68^ Wu et al.^69^ found decreased levels of m^6^A in bovine mammary epithelial cells treated with AFB1, coupled with increased expression of the demethylase ALKBH5 and decreased expression of methyltransferases METTL3 and METTL14. However, how these changes regulate cellular response to AFB1 damage is unknown.^69^ In another study, when C57BL/6J mice were fed a diet of 600 μg/kg AFB1 over 4 weeks, they showed higher levels of m^6^A modification in the liver as well as decreased expression of FTO and YTHDF2, and higher expression of METTL3.^70^ The authors found that treatment with resveratrol, a naturally occurring antioxidant, could attenuate increased m^6^A levels in the liver and suggested that this may be a mechanism of promoting antioxidant gene expression to repair hepatic function.^70^ Another study showed that AFB1 exposure decreases the m^1^A writer TRMT6 in HepaRG cells.^71^ Furthermore, human colon Caco‐2 cells exposed to a chemical precursor of AFB1 called Versicolorin A exhibited increased expression of the m^5^C reader ALYREF.^72^

#### 3.2.5 CNTs

CNTs are composed of graphene sheets rolled into a cylindrical fiber and produce particulate matter similar to that of asbestos.^73^ A type of CNT, a multiwalled CNT (MWCNT) called Mitsui‐7, was classified as possibly carcinogenic to humans (Group 2B) in 2014.^74^ One study found that bronchial epithelial cells exposed to MWCNTs had decreased global m^6^A methylation and increased levels of 5‐hydroxymethylcytosine (hm^5^C) on RNA.^75^ Additionally, exposure to single‐walled CNTs showed higher levels of hm^5^C on RNA.^75^ Another study showed that mice exposed to low, medium, and high doses of MWCNT had upregulated levels of the m^1^A writer TRMT61A.^76^

#### 3.2.6 CYP

CYP is an antineoplastic and alkylating agent used to treat certain cancers and diseases.^77^ CYP has been classified as an IARC Group 1 carcinogen and has been found to cause bladder cancer and acute myeloid leukemia.^78^ A study of the effects of CYP on cardiotoxicity showed that rat neonatal cardiomyocytes treated with CYP for 2 days had increased m^6^A levels and increased METTL3 expression.^79^ METTL3‐mediated m^6^A on junctophilin‐2 (*Jph2*) mRNA decreased *Jph2* expression and promoted cardiac dysfunction under cardiotoxic stress.^79^ In another study, treatment of normal human ovarian granulosa cells (hGCs) or mouse ovaries with CYP also showed increased levels of m^6^A in a time‐ and concentration‐dependent manner.^80^ CYP‐treated hGCs and mouse ovaries also showed increased expression of METTL3, METTL14, ZC3H13, and KIAA1429, and decreased expression of FTO and the readers YTHDF1, YTHDF2, YTHDC1, and YTHDC3, in a time‐dependent manner.^80^ The authors suggest that these effects may have a role in CYP‐induced ovarian damage.^80^

#### 3.2.7 Endocrine‐disrupting chemicals: Polychlorinated biphenyls (PCBs), Di(2‐ethylhexyl)phthalate (DHEP), triclosan, and bisphenol‐A (BPA)

PCBs are synthetic organic compounds that were widely used in dielectric fluids and sealants until 1979 and can contaminate soil, air, water, and food.^81^ PCBs are IARC Group 1 carcinogens associated with melanoma, non‐Hodgkin's lymphoma, and breast cancer.^81^ Aluru et al.^82^ exposed zebrafish embryos to 10 nM PCB126 for 7 h and found 15 different RNA transcripts with m^6^A methylation. Interestingly, these 15 RNA transcripts were associated with AHR signaling, which is involved in responses to environmental stress.^82^ In a study of toxicant‐associated steatohepatitis, C57BL/6J male mice exposed to PCB126 and a high‐fat diet (HFD) had altered levels of 10 types of RNA modifications in total RNA.^83^ In particular, mice fed HFDs and exposed to a PCB mixture showed increased m^6^Am and PCIF1 expression.^83^ Furthermore, a study of transcriptional profiling in response to mixed PCB exposure in primary blood mononuclear cells showed decreased expression of the m^6^A writers WTAP and RBM15.^84^

DHEP is an IARC group 2B carcinogen and Zhao et al.^85^, ^86^ showed that exposure to DHEP in Sprague–Dawley rats resulted in increased m^6^A levels, increased YTHDC2 expression, and decreased FTO expression. In addition, DHEP exposure resulted in decreased NRF2 signaling and increased oxidative stress, which promoted testicular toxicity.^86^ The carcinogenicity of triclosan, BPA, and vinclozolin is still under consideration.^87^, ^88^, ^89^ In a study of nonalcoholic fatty liver disease, zebrafish larvae treated with BPA or triclosan showed decreased global m^6^A levels.^90^ Both triclosan and BPA exposures showed increased expression of YTHDC1 and YTHDF1, whereas only triclosan exposure showed increased FTO expression.^90^ Furthermore, another study found that exposure of A549 cells to BPA or vinclozolin similarly showed decreased m^6^A levels.^44^

### 3.3 Inhalation exposures

There is evidence to suggest that cigarette smoke (CS) exposure can lead to the development of lung cancer, pancreatic ductal adenocarcinoma (PDAC), and esophageal squamous cell carcinoma (ESCC), through changes in RNA modifications. Studies have also found that particulate matter in outdoor air pollution can affect RNA modifications, but it is unclear whether this particulate matter can contribute to carcinogenesis.

#### 3.3.1 CS

As of 2019, 1.14 billion individuals worldwide were estimated to be tobacco smokers.^91^ Tobacco smoke is an IARC Group 1 carcinogen with associations with a variety of cancer types including lung, esophagus, and pancreatic cancer.^92^ Tobacco is most commonly smoked as cigarettes and secondhand exposure to tobacco smoke or involuntary smoking is also considered a Group 1 carcinogen.^92^ Jin et al.^93^ showed that A549 cells exposed to CS condensate (CSC) had increased m^6^A levels (Table 2).^93^ Gene‐specific m^6^A increases were found on the transcript of threonine/serine kinase *Dapk2*, a tumor suppressor in nonsmall cell lung cancer (NSCLC).^93^ In particular, upregulated METTL3 expression increased m^6^A on the *Dapk2* transcript, resulting in decreased *Dapk2* mRNA stability and expression through a YTHDF2‐dependent mechanism.^93^ Decreased DAPK2 resulted in downstream activation of oncogenic nuclear factor‐κB (NF‐κB) signaling, establishing a role for the METTL3‐DAPK2‐NF‐κB axis in promoting CSC‐induced NSCLC malignancy.^93^

**Table 2:** Role of RNA modifications during CS‐induced carcinogenesis

Exposure form	Dose	Cells or organisms	Cancer type	Finding	Proposed	References
					pathway	
CS condensate	100 μg/ml	A549 cells	NSCLC	↑m^6^A, ↑METTL3	METTL3‐DAPK2‐NF‐κB	^93^
CS extract	2%	HBE cells	Lung cancer	↑m^6^A, ↑METTL3	METTL3‐ YTHDF2‐ZBTB4	^94^
CS	20%	BEAS‐2B cells	Lung cancer	↓YTHDC2	YTHDC2‐CYLD‐NF‐κB	^95^
CS condensate	100 μg/ml	HPDE6‐C7 cells	PDAC	↑METTL3	METTL3‐miR‐25‐3p‐PHLPP2‐AKT	^96^
CS condensate	100 μg/ml	Het1A cells	ESCC	↑ALKBH5	YTHDF1‐YY1BM‐eEF2K	^97^
CS	–	Human subjects	–	↓m^6^A, ↑METTL3	–	^98^

Abbreviations: ALKBH5, Alkb homolog 5; CS, cigarette smoke; ESCC, esophageal squamous cell carcinoma; HBE, human bronchial epithelial; m^6^A, *N* ^6^‐methyladenosine; METTL3, methyltransferase‐like family member 3; NSCLC, nonsmall cell lung cancer; PDAC, pancreatic ductal adenocarcinoma; YTHDC2, YTH domain‐containing 2.

Similarly, in HBE cells transformed with 2% CS extract, increased levels of m^6^A and upregulated expression of METTL3 were observed after 48 h of treatment.^94^ In particular, METTL3‐mediated increases of m^6^A on the 3′‐UTR of the tumor suppressor *Zbtb4* resulted in decreased ZBTB4 expression through YTHDF2‐mediated mRNA decay.^94^ As a result, decreased ZBTB4 expression promoted CS‐induced epithelial‐mesenchymal transition and carcinogenesis.^94^ In another study, HBE (BEAS‐2B) cells exposed to CS had downregulated expression of YTHDC2.^95^ Mechanistically, YTHDC2 was found to bind m^6^A sites on the transcript of the tumor suppressor *Cyld* and promoted *Cyld* mRNA stability.^95^ As CYLD normally inhibits the NF‐κB pathway, decreased YTHDC2 expression after CS exposure can promote cancer cell proliferation by modulating the CYLD/NF‐κB axis in CS‐induced lung cancer.^95^

In a PDAC study, exposure to 100 μg/ml of CSC in human pancreatic duct epithelial (HPDE6C7) cells for 48 h increased the expression of METTL3 and the microRNA (miRNA) miR‐25‐3p.^96^ METTL3 promoted the maturation of the miRNA miR‐25‐3p in an m^6^A‐dependent manner and the NF‐κB‐associated protein NKAP served as a reader of m^6^A on miR‐25‐p3.^96^ Given that miR‐25‐3p has a role in inhibiting PHLPP2 and activating AKT signaling in cancer progression, the study suggests a role of the METTL3‐miR‐25‐3p‐PHLPP2‐AKT axis in promoting cigarette smoking‐induced PDAC tumorigenesis.^96^

A study in cigarette smoking‐induced ESCC showed that the translation of a long noncoding RNA (*LINC20078*) into the micropeptide (YY1BM) was regulated by m^6^A modifications and mediated by YTHDF1.^97^ In ESCC, YY1BM suppresses transcription of an oncogenic factor, EEF2K, to slow cancer progression.^97^ CS exposure was found to upregulate ALKBH5, resulting in the demethylation of *LINC20078* and reduced YY1BM levels, ultimately promoting ESCC carcinogenesis.^97^

Finally, one study utilized data from the Beijing Truck Driver Air Pollution Study to correlate smoking exposure to m^6^A modification in human subjects.^98^ Long‐term smoking was correlated with a 10.7% decrease in global m^6^A levels in blood leukocytes compared to nonsmokers, whereas acute smoking exposure did not correlate with changes in m^6^A.^98^

#### 3.3.2 Particulate matter

Particulate matter in outdoor air pollution is an IARC Group 1 carcinogen based on evidence that exposure can cause lung cancer.^99^ This group includes fine inhalable particles with diameters smaller than 2.5 μm, referred to as PM2.5, and can comprise inorganic ions, metal oxides, and carbonaceous material.^99^ HBE and A549 cells exposed to 62.5, 125, and 250 μg/ml of PM2.5 showed increases in m^6^A levels.^100^ In particular, METTL3 promoted the mRNA stability of cell death regulator, OSGIN1, in an m^6^A‐dependent manner, resulting in increased apoptosis, cell cycle arrest, and autophagy in PM2.5‐exposed cells.^100^ Similarly, another study showed that the lungs of C57BL.6J male mice exposed to PM2.5 had increased levels of m^6^A and increased expression of METTL3 and METTL14.^101^ Interestingly, this effect could be reversed after exposure to purified air.^101^

In contrast, a study in A549 cells exposed to >62 μg/ml of PM for 24 or 48 h showed decreased global levels of m^6^A.^44^ The authors also reviewed a data set of human participants exposed to high levels of PM2.5 and found increased expression of the writers METTL3 and WTAP, the erasers FTO and ALKBH5, and the reader HNRNPC in leukocyte cells.^44^ In a study of differentially expressed genes in HBE cells after exposure to 50 μg/ml PM2.5, the m^6^A reader HNRNPA2B1 was upregulated and the m^5^C reader ALYREF was downregulated.^102^ Lastly, a study of pulmonary fibrosis noted increased mRNA and protein levels of the m^5^C writer NSUN2 in the lungs of C57BL/6 male mice exposed to an average of 60 μg/ml of PM2.5 for 12 weeks.^103^

### 3.4 Dermal exposures

Evidence indicates that UV exposure can promote carcinogenesis through RNA modifications. In contrast, exposure to γ‐radiation can modulate RNA modification and regulatory enzymes, but more research is needed to determine how this could promote carcinogenesis.

#### 3.4.1 UV radiation

Natural sources of UV exposure include solar radiation (UVA and UVB).^104^ Solar UV radiation, as well as artificial sources of radiation such as tanning beds, can cause melanoma and other skin cancers.^104^ Yang et al.^105^ showed that in human keratinocytes exposed to UVB irradiation, METTL14 was downregulated and levels of m^6^A were decreased. Mechanistically, METTL14 levels were decreased through UVB‐induced NBR1‐dependent selective autophagy.^105^ In response to UVB‐induced damage, METTL14 was found to regulate translation of the genome repair factor DDB2 through an m^6^A/YTHDF1‐dependent mechanism.^105^ Furthermore, skin‐specific heterozygous deletion of METTL14 in mice accelerated UVB‐induced carcinogenesis, suggesting that METTL14 suppresses tumorigenesis by promoting global genome repair.^105^

Other research has shown that m^6^A modification transiently accumulates at sites of DNA damage. METTL3, METTL14, and FTO were also found to localize to sites of DNA damage in response to UVA laser micro‐irradiation or UVC irradiation in U2OS cells, A375 melanoma cells, and HeLa cells.^106^ Additionally, the DNA damage repair factor PARP1 was found to recruit METTL3 and METTL3‐mediated RNA methylation promoted DNA repair by recruiting DNA polymerase Polκ to damaged sites.^106^ There is also evidence that METTL16 accumulates at UVA‐radiated lesions and methylates snRNAs during later stages of genome repair.^107^ UVA radiation was also found to decrease global m^1^A modification levels.^107^ In addition, it has been shown that UVB exposure can rapidly reduce RNA levels of the m^5^C writer *Nsun2* in human epidermal and dermal cells.^47^

#### 3.4.2 γ‐Radiation

Exposure to γ‐radiation can occur through natural sources and medical use, and can lead to leukemia, breast cancer, and thyroid cancer, among others.^108^ Bone marrow cells from C57BL/6J mice exposed to γ‐irradiation showed reduced m^6^A levels after 2 h, accompanied by increased expression of METTL14 and ALKBH5, and decreased expression of METTL3, WTAP, and FTO.^109^ m^6^A modifications were suggested to play a role in the development of radiation toxicity, and inhibition of FTO and ALKBH5 reduced γ‐radiation hematopoietic injury.^109^

### 3.5 Viral infections

Viruses can modulate RNA modifications in both viral and host RNAs to enhance replication, promote long‐term latency, and regulate life cycles.^15^ Research suggests that Hepatitis B, Epstein–Barr virus (EBV), and Kaposi's sarcoma‐associated herpesvirus (KSHV) are all oncogenic viruses that can modulate the host's RNA modifications to promote carcinogenesis.^15^

#### 3.5.1 Hepatitis B virus (HBV)

**Figure 3:**
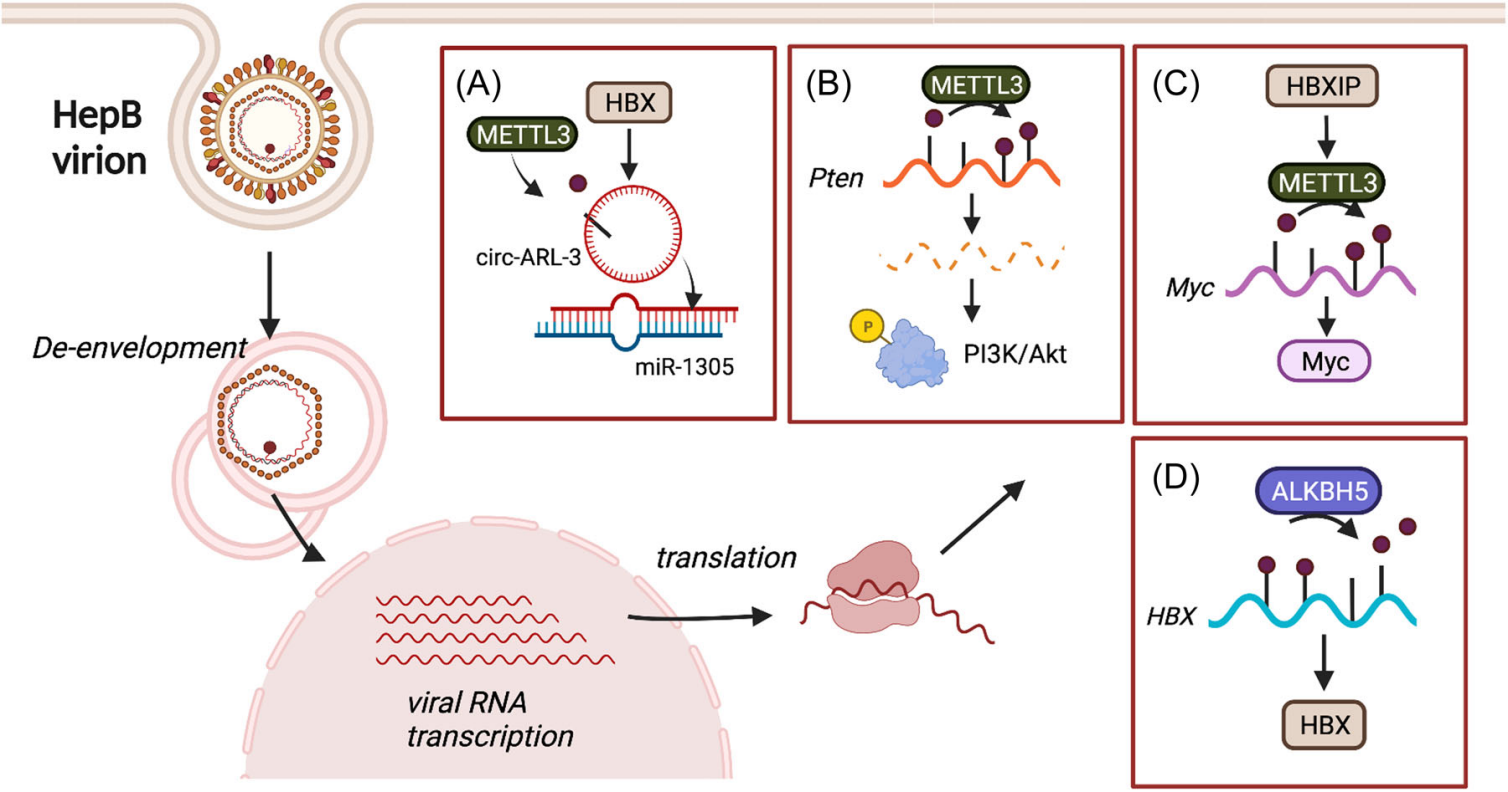
Pathways of carcinogenesis during hepatitis B infection. (A) Viral protein HBX upregulates m^6^A modification of circ‐ARL‐3 to increase its expression, leading to inhibition of tumor suppressor miR‐1305. (B) Upregulation of methyltransferase‐like family member 3 (METTL3) leads to degredation of *Pten* transcripts and activation of phosphatidylinositol 3‐kinase (PI3K)/AKT pathway. (C) Viral protein hepatitis B X‐interacting protein (HBXIP) upregulates METTL3 to promote increased translation of oncogene Myc. (D) Upregulation of Alkb homolog 5 (ALKBH5) leads to increased production of oncogenic HBX.^120^

HBV is an IARC Group 1 carcinogen.^110^ Chronic infection with HBV can cause HCC and cholangiocarcinoma.^110^ Kim et al.^111^ found that in primary human hepatocytes, infection with HBV induced differential m^6^A changes in various host RNAs (Figure 3). In particular, HBV infection increased METTL3‐mediated m^6^A modification on the tumor suppressor *Pten* transcript, resulting in decreased *Pten* mRNA stability.^111^ Decreased PTEN expression resulted in the activation of the PI3K/AKT pathway, contributing to HBV‐mediated HCC carcinogenesis.^111^

In addition, HBX, a Hepatitis‐B viral protein and a major etiological factor in HCC,^112^ has been shown to promote m^6^A modification of a circRNA, circ‐ARL3, by upregulating METTL3 expression.^113^ As a result, increased circ‐ARL3 could then bind an HCC tumor suppressor miRNA, miR‐1305, and promote HBV‐related HCC pathogenesis.^113^ Additionally, Qu et al.^114^ showed that HBV could upregulate ALKBH5 through an HBX‐WDR5‐H3K4me3 feedback loop, causing ALKBH5 to be highly expressed in HBV‐induced HCC. Additionally, ALKBH5 promoted *Hbx* mRNA stability by decreasing m^6^A modification, suggesting an oncogenic role for ALKBH5 in HBV‐induced HCC.^114^

Furthermore, HBV infection is also associated with gastric cancer development, and a study showed that the hepatitis B X‐interacting protein could upregulate METTL3 during HBV infection.^115^ Mechanistically, METTL3‐mediated m^6^A modification on the proto‐oncogene *c‐Myc* transcript resulted in increased MYC expression and gastric cancer progression.^115^

#### 3.5.2 EBV

EBV was the first oncogenic virus discovered, and is an IARC Group 1 carcinogen that causes Burkitt's lymphoma and Hodgkin's lymphoma, among others.^110^ Lang et al.^116^ showed that EBV‐infected cells had elevated levels of METTL14. METTL14 was also found to colocalize with the EBV latent antigen EBNA3C.^116^ Increased METTL14 upregulated the expression of EBV latent antigens, including EBNA3C, in an m^6^A‐dependent manner, establishing a positive feedback loop that contributed to EBV‐induced oncogenesis.^116^ Xia et al.^117^ showed that YTHDF1 could repress EBV replication by degrading m^6^A‐modified viral transcripts through recruitment of ZAP, DDX17, and DCP2, making it a potential therapeutic target for EBV‐associated cancers.^117^

#### 3.5.3 KSHV

KSHV is a γ‐2 herpesvirus and an IARC Group 1 carcinogen that can cause Kaposi's sarcoma and primary effusion lymphoma.^110^ Tan et al.^118^ analyzed m^6^A and m^6^Am modifications together, and noted that in the host RNA of cells latently infected with KSHV, 5′‐UTR were hypomethylated and 3′‐UTR were hypermethylated compared with uninfected cells. These differentially methylated genes belonged to pathways involved in cellular transformation, including mTOR signaling, ephrin receptor signaling, and hypoxia, suggesting that KSHV may utilize RNA modifications to induce tumorigenesis.^118^ In addition, m^6^A has been shown to be required for the onset of KSHV lytic replication, a major contributor to cancer development, and regulate pre‐mRNA splicing of the replication transcription factor.^119^

## 4 CONCLUSIONS

Compelling evidence has demonstrated the crucial role of RNA modifications in maintaining the oncogenic traits of established cancers and cancer cells. Recent emerging evidence suggests that upon exposure to external carcinogens, RNA modifications are also implicated much earlier in the carcinogenic process. In this review, we summarize advances in the field, and consider how various exogenous exposures may use RNA modifications to promote oncogenesis.

For some carcinogens such as arsenic and CS, there are several described mechanisms by which RNA modifications promote carcinogenesis. More studies must be conducted to understand how the various mechanisms co‐exist and interact with one another to pinpoint where potential therapies may be most successful. However, for many carcinogens such as CNTs and γ‐radiation, a direct link to carcinogenic processes is yet to be established and the limited evidence presented here indicates that these would be areas worth pursuing.

Notably, the existing research on RNA modifications in carcinogenesis focuses heavily on m^6^A modifications and their writers, erasers, and readers. However, preliminary evidence for exposures to carcinogens such as UV exposure and PM suggests that m^5^C and m^1^A modifications may also play an important role in carcinogenesis. As research into RNA modifications continues to gain prominence, further studies must investigate how different types of chemical modifications in RNA affect carcinogenic processes. Finally, out of 121 Group 1 IARC carcinogens, only 16 were described in this review, indicating a large breadth of potential research into other carcinogenic exposures that are yet to be fully explored. Overall, the role of RNA modifications in carcinogenesis is a new emerging field for continuing to elucidate the molecular mechanisms of oncogenesis and for developing new cancer therapies.

## AUTHOR CONTRIBUTIONS

Yu‐Ying He conceived the project. Michelle Verghese, Emma Wilkinson, and Yu‐Ying He wrote the paper.

## Data Availability

Data from this study are available from the corresponding author upon request.
